# A Comprehensive Analysis and Splicing Characterization of Naturally Occurring Synonymous Variants in the *ATP7B* Gene

**DOI:** 10.3389/fgene.2020.592611

**Published:** 2021-02-25

**Authors:** Xiaoying Zhou, Wei Zhou, Chunli Wang, Lan Wang, Yu Jin, Zhanjun Jia, Zhifeng Liu, Bixia Zheng

**Affiliations:** ^1^Department of Gastroenterology, Children’s Hospital of Nanjing Medical University, Nanjing, China; ^2^Nanjing Key Laboratory of Pediatrics, Children’s Hospital of Nanjing Medical University, Nanjing, China

**Keywords:** Wilson disease, ATP7B gene, minigene assays, splicing aberrations, synonymous mutations

## Abstract

Next-generation sequencing is effective for the molecular diagnosis of genetic diseases. However, the identification of the clinical significance of synonymous variants remains a challenge. Our previous study showed that some synonymous variants in *ATP7B* gene produced splicing disruptions, leading to Wilson disease (WD). To test the hypothesis that synonymous variants of *ATP7B* cause abnormal splicing by disrupting authentic splice sites or splicing regulatory elements, we used computational tools and minigene assays to characterize 253 naturally occurring *ATP7B* gene synonymous variants in this study. Human Splicing Finder (HSF) and ESE Finder 3.0 were used to predict the impact of these rare synonymous variants on pre-mRNA splicing. Then, we cloned 14 different wild-type Minigene_ATP7B_ex constructs for *in vitro* minigene assay, including 16 exons of *ATP7B* gene. After computational prediction, 85 candidate variants were selected to be introduced into the corresponding Minigene_ATP7B_ex constructs for splicing assays. Using this two-step procedure, we demonstrated that 11 synonymous variants in ExAc database (c.1620C>T, c.3888C>T, c.1554C>T, c.1677C>T, c.1830G>A, c.1875T>A, c.2826C>A, c.4098G>A, c.2994C>T, c.3243G>A, and c.3747G>A) disrupted RNA splicing *in vitro*, and two (c.1620C>T and c.3243G>A) of these caused a complete exon skipping. The results not only provided a reliable experimental basis for the genetic diagnosis of WD patients but also offered some new insights into the pathogenicity of synonymous variants in genetic diseases.

## Introduction

Wilson disease (OMIM, #277900, WD) is an autosomal, recessively inherited disorder characterized by the dramatic accumulation of intracellular hepatic copper with subsequent hepatic and neurologic abnormalities (Xie and Wu, 2017). The worldwide prevalence of Wilson disease is estimated to be on the order of 30 per one million, with a gene frequency of 0.56% and a carrier frequency of one in 90 (Sandahl et al., 2019). Loss-of-function mutations in ATPase copper-transporting beta gene (*ATP7B*) are responsible for WD. The *ATP7B* gene (OMIM, ^∗^606882) is located on chromosome 13q14.3 encoding copper-transporting ATPase 2 (ATP7B), acting as a plasma membrane copper-transport protein. Liver disease, which shows a high variability in patients, is the main clinical manifestation of WD, with or without neurological and/or psychological symptoms and Kayser–Fleischer corneal rings (Walshe, 2017). Due to the heterogeneity of the clinical features and the age of onset, WD is prone to be misdiagnosed or prolonged in the diagnosis cycle (Balashova et al., 2019). At present (Hedera, 2019), genetic screening of *ATP7B* gene is widely used in the diagnosis of WD, which can greatly benefit the clinical diagnosis and management of patients (Li et al., 2019). However, synonymous variants yield from mutation screening with uncertain clinical significance (VUS). Thus, the functional and clinical interpretations pose a challenge for genomic diagnosis of WD.

Up to now, a total of 995 mutations in *ATP7B* were annotated in the Human Gene Mutation Database (HGMD)^1^, in which 67 splice variants located in the non-coding region were included. No synonymous variants were reported in the HGMD. Synonymous variants are often considered to be neutral because they do not change the amino acid coding. However, recent studies have shown that synonymous/missense mutations can lead to abnormal splicing of mRNA by disrupting splicing regulatory elements (SREs), which act as a new, gradually recognized pathogenic mechanism in genetic diseases. This mechanism has been reported in Gitelman syndrome, progressive familial intrahepatic cholestasis, Netherton syndrome, and other genetic disorders (Byrne et al., 2009; Dal Mas et al., 2015; Takeuchi et al., 2015). Our previous study demonstrated that the synonymous variant (c.4014T>A) and missense variant (c.2755C>G/p.R919G) in WD patients resulted in exon skipping in pre-mRNA splicing, leading to protein truncation and WD (Wang et al., 2018).

The recognition of intron and exon boundaries by spliceosome is one of the key steps in the splicing process [9]. Exonic splicing enhancers (ESEs) and exonic splicing silencers (ESSs) are SREs located in the coding region. ESEs and ESSs are *cis*-acting elements and are composed of six to eight nucleotides. After binding to the ESEs, the SR protein can promote splicing by recruiting U1 snRNP to the donor splicing site or balancing the inhibitory effect of ESSs [10]. Human-derived SR proteins include SF2/ASF, SC35, SRp40, and SRp55, also known as SRSF1, SRSF2, SRSF5, and SRSF6, respectively (Claverie-Martin et al., 2015). ESE Finder 3.0 can recognize ESE motifs from the point of view of SR proteins, and this method can predict the disrupted SR binding protein by variation (Cartegni and Krainer, 2002). The hnRNP protein binds to ESSs, which can inhibit the binding of the spliceosomal complexes to the splicing site and block the combination of SR protein and ESEs (Cartegni et al., 2003). Donor or acceptor splice sites are the most conservative splicing sequence, which is recognized by different components of spliceosome in the process of splicing. Donor and acceptor splice sites were initially recognized by U1 snRNP and U2 auxiliary factor (U2AF), respectively, then formed complexes [13]. Synonymous mutations lead to changes in one or more of the above-mentioned splicing factors, which may lead to mRNA splicing abnormalities (Singh RnandSingh, 2018).

To test the hypothesis that synonymous variants of *ATP7B* cause abnormal splicing by disrupting authentic splice sites or splicing regulatory elements, we used computational tools and minigene assays to characterize 253 synonymous variants in the Exome Aggregation Consortium (ExAc) database and literature. There were 14 kinds of wild-type plasmids constructed by inserting exons into the splicing vector pSPL3, covering 16 exons (except exons 1, 2, 8, 9, and 21). A total of 85 candidate variants were investigated by minigene assays *in vitro*, 11 of which were shown to cause abnormal pre-mRNA splicing of *ATP7B*.

## Materials and Methods

### Variant Inclusion and Computational Tools

By searching the ExAc database^2^, which is incorporated into the gnomad database now^3^, 247 rare synonymous variations of *ATP7B* gene with allele frequencies less than 0.005 were included. The rare synonymous variations of *ATP7B* reported in the literature were also reviewed, and finally six more variants were also included. The list of the 253 synonymous variations is shown in Supplementary Table 1. Human Splicing Finder (HSF)^4^ was used to analyze the effect of these variants on the splicing of pre-mRNA. ESE Finder 3.0^5^ was further applied to analyze variations that were predicted to affect ESEs (threshold is SRSF1: 1.956, SRSF2: 2.383, SRSF5: 2.67, SRSF6: 2.676).

### Construction of Wild-Type Minigene_ATP7B_ex Plasmids

We used pSPL3 vector, a generous gift from Dr. Irene Bottillo (Sapienza University of Rome, Italy) and Dr. Leping Shao (Qingdao University, China). The exons and flanking introns of the *ATP7B* gene were amplified by Phanta^®^ Super-Fidelity DNA Polymerase (Vazyme Biotech Co., Ltd., China) with the primers indicated in Supplementary Table 2. The primers used in this step were edited by Primer 5 software, and the corresponding viscous terminal sequence was added to the end. pSPL3 vector was cut with XhoI and BamHI (Invitrogen, United States). All of the indicated fragments were cloned into a pSPL3 vector with the XhoI and BamHI using ClonExpressTM II One Step Cloning Kit (Vazyme Biotech Co., Ltd., China). All constructs were sequenced to confirm by Sanger sequencing (TsingKe Co., Ltd., China), and the corresponding exon wild-type plasmids (Minigene ATP7B_ex) were screened. All clones were functionally checked in 293T cells (Cell Bank of the Chinese Academy of Sciences, China., Cat# GNHu17), and 14 kinds of wild-type plasmids were constructed by inserting exons into the splicing vector pSPL3, covering 16 exons (except exons 1, 2, 8, 9, and 21). The pattern for constructing the plasmids can be found in Supplementary Figure 1. The sequencing data of the RT-PCR products of the 14 kinds of wild-type plasmids which covered 16 exons of ATP7B are shown in Supplementary Figure 2.

### Site-Directed Mutagenesis

Based on the construction of wild-type Minigene ATP7B_ex, various mutant Minigene ATP7B_ex capture plasmids were constructed by PCR-mediated *in vitro* site-directed mutagenesis using Mut Express MultiS Fast Mutagenesis Kit V2 (Vazyme Biotech Co., Ltd., China). The design of the 85 candidate DNA site-directed mutagenesis primers for synonymous mutations is shown in Supplementary Table 3. The primers used in this step were edited by Primer 5 software. The corresponding wild-type Minigene_ATP7B_ex was amplified by PCR, with the primers shown in Supplementary Table 3, to form a linear mutant plasmid and then reorganized. The gene sequences were confirmed by direct sequencing.

### Transfection of Eukaryotic Cells and RT-PCR of Minigenes

293T cells were seeded in 12-well plates and grown in 1 ml Dulbecco’s Modified Eagle’s Medium (10% fetal bovine serum). After the cells were 50–70% confluent, 293T cells were transfected with 1 μg of DNA (containing either wild-type or mutant Minigene ATP7B_ex) using Lipofectamine 2000 (Invitrogen, America). At 24 h after transient transfection, total RNA was extracted from cells using TRIzol (Takara, Japan). The first cDNA strand was reversely transcribed using the Superscript III Reverse Transcriptase (Invitrogen, America) together with random hexanucleotide primers. Then, 2 μl of each cDNA was amplified by TaKaRa Ex Taq (Takara, Japan), with primers located in the two cassette exons of the pSPL3 vector. The primer sequences were as follows: forward SD 5′-TCTGAGTCACCTGGACA ACC-3′ and reverse SA 5′-ATCTCAGTGGTATTTGTGAGC-3′. The PCR products were analyzed by agarose gel electrophoresis and proven by sequencing of the extracted DNA. Gel containing 1.5% agarose in Tris-acetate-EDTA buffer was prepared and applied. The marker used in the agarose gels was DL2000 Plus DNA Marker (Vazyme, China). The ratio of normal and abnormal transcripts was analyzed by grayscale value, and the experiment was repeated four times in each group. The mean value was obtained.

## Results

### Computational Analysis of Synonymous Variations in *ATP7B*

**FIGURE 1:**
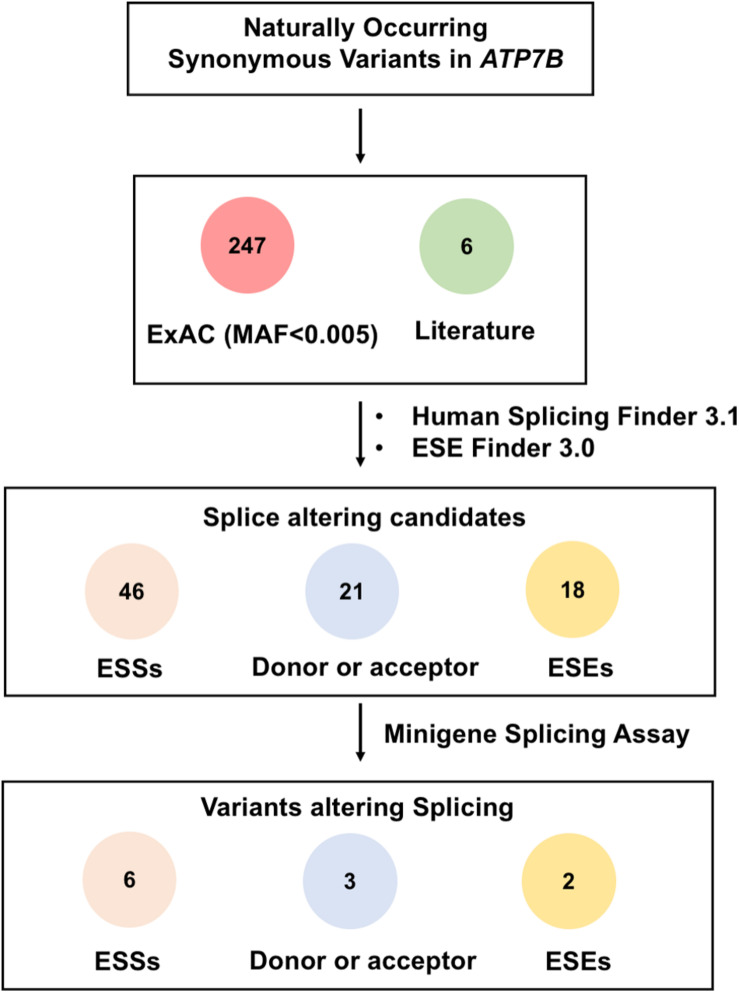
Schematic overview of prioritization and functional assessment of 253 naturally occurring synonymous variations in *ATP7B* gene. A total of 253 rare synonymous variants that presented in ExAc database or previously reported in literature were included in this study. Based on the predicted results of Human Splicing Finder 3.1 and ESE Finder 3.0, 46 variants were predicted to create ESS motifs, 21 variants would affect donor or acceptor splice sites, and 18 variants were likely to disrupt the binding of ESEs with two or more SR proteins. Then, all 85 candidate variants were screened by minigene splicing assays. As a result, 11 variants were confirmed to disrupt the splicing of pre-mRNA: Six variants were predicted to create ESS motifs, three variants would affect donor or acceptor splice sites, and two variants were predicted to disrupt ESE motifs.

A total of 253 rare synonymous variants that presented in ExAc database or were previously reported in literature were included in this study (El-Mougy et al., 2014; Dong et al., 2016; Qian et al., 2019). The allele frequencies of these variants are all less than 0.005 and not yet functionally investigated. To select possible spliceogenic variants, we firstly prioritized the variants using a computational algorithm HSF. Based on the predicted results of HSF, 46 variants were likely to create ESSs motifs, and 21 variants would affect donor or acceptor splice sites. What is more, 140 variants were likely to disrupt the ESEs. Previous studies showed that ESE variants that affected the binding of two or more SR proteins are more probable to produce splicing disruption (Takeuchi et al., 2015). Therefore, further prediction using ESE Finder 3.0 was carried out for these 140 variants, of which 18 variants that predicted to disrupt two or more SR proteins were screened in minigene assays (Supplementary Table 2). A flow chart in Figure 1 shows the two-step screening of all synonymous variants.

### Minigene Assays of 85 Synonymous Variants in *ATP7B*

We constructed 14 different wild-type Minigene_ATP7B_ex plasmids, including 16 exons of *ATP7B* gene. According to the results of computational calculation, we divided the 85 synonymous mutations into the following three groups for Minigene Assays: (1) 18 variants that may destroy a variety of ESEs, (2) 46 variants that may produce new ESSs, and (3) 21 variants that may affect donor or acceptor site splicing. Taken together, 85 variants were introduced into their corresponding Minigene_ATP7B_ex and assayed in 293T cells. As a result, 11 variants were confirmed to disrupt the splicing of pre-mRNA, which were divided into three groups as described below. The results of the computational calculation and minigene assays of the 11 variants are detailed in Table 1.

**TABLE 1:** Computational prediction and splicing outcome of 11 synonymous variants in the *ATP7B* gene.

**Number**	**DNA**	**RNA**	**Protein**	**Bioinformatics**	**Splicing outcome**
1	c.1554C>T	r.1544_1707del	p.(Gly515Aspfs*37)	[+] ESS	Exon 4 skipping (58.15%)
2	c.1620C>T	r.1544_1707del	p.(Gly515Aspfs*37)	[−] ESE (SRSF1, SRSF5)	Exon 4 skipping (100.00%)
3	c.1677C>T	r.1544_1707del	p.(Gly515Aspfs*37)	[+] ESS	Exon 4 skipping (16.79%)
4	c.1830G>A	r.1708_1869del	p.(Ile570_Glu623del)	[+] ESS, new acceptor site	Exon 5 skipping (17.40%)
5	c.1875T>A	r.1870_1946del	p.(Glu624Valfs*103)	[+] ESS, new acceptor site	Exon 6 skipping (35.77%)
6	c.2826C>A	r.2731_2865del	p.(Ala911_Pro955del)	[+] ESS, new acceptor site	Exon 12 skipping (21.44%)
7	c.2994C>T	r.2989_3060del	p.(Val997_Lys1020del)	New donor site, [+] ESS	Exon 13 del 72 bp (57.79%)
8	c.3243G>A	r.3061_3243del	p.(Ile1021_Glu1081del)	Altering WT donor site	Exon 14 skipping (100%)
9	c.3747G>A	r.3701_3904del	p.(Val1234_Arg1301del)	New acceptor site	Exon 18 skipping (34.93%)
10	c.3888C>T	r.3701_3904del	p.(Val1234_Arg1301del)	[−] ESE (SRSF1, SRSF6), [+] ESS	Exon 18 skipping (25.00%)
11	c.4098G>A	r.4023_4125del	p.(Gly1341Alafs*80)	[+] ESS	Exon 20 skipping (14.79%)

*[+], creation of splicing motifs; [−] breakage.*

### Evaluation of 18 Synonymous Variants Predicted to Eliminate ESE Motif

**FIGURE 2:**
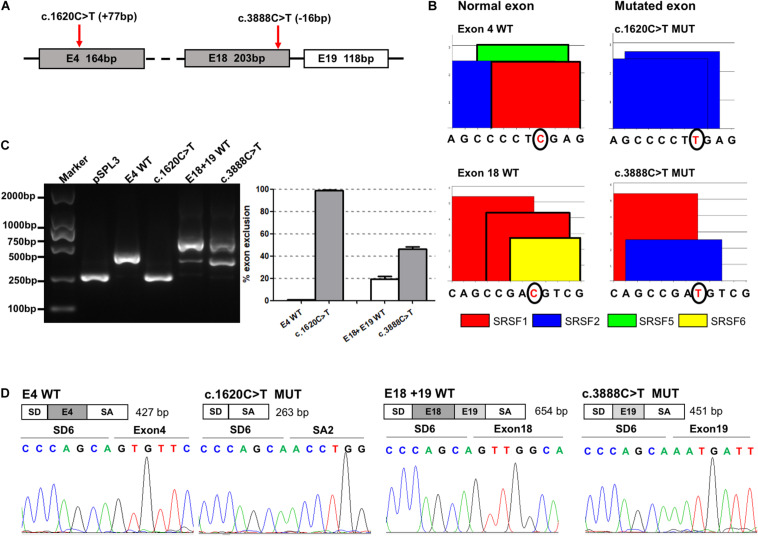
The effect of variants c.1620C>T and c.3888C>T on *ATP7B* pre-mRNA splicing. **(A)** The exonic localization of the c.1620C>T and c.3888C>T variants in the *ATP7B* gene. **(B)** ESE Finder 3.0 predicted that c.1620C>T would destroy ESEs binding to SRSF1 and SRSF5 and c.3888C>T would destroy ESEs binding to SRSF1 and SRSF6. **(C)** Agarose electrophoresis of RT-PCR products is shown on the left and the grayscale value analysis on the right. The RT-PCR results shows that the c.1620C>T mutant resulted in exon 4 (164 bp) being skipped completely, and the c.3888C>T mutant increased the proportion of abnormal splicing by 25% compared with the wild type. All data are represented as mean ± SEM (*n* = 4). Quantification of the aberrant splicing percentage in the graph was calculated by densitometric analyses of the gel-fractionated RT-PCR products as the percentage of exclusion (%) = [MUT band/(MUT band + WT band)] × 100. **(D)** Sequencing chromatograms of the RT-PCR products amplified from the wild type and mutants. WT, wild type; MUT, mutant.

There were 18 selected synonymous variations verified by *in vitro* minigene assays. Our results showed that c.1620C>T and c.3888C>T induced skipping of exons 4 (100%) and 18 (25%), respectively (Figure 2). Figure 2A shows the exact location of these two variants in the *ATP7B* gene. ESE Finder 3.0 predicted that the c.1620C>T would break ESEs binding to SRSF1 and SRSF5, and the c.3888C>T would disrupt the ESEs that bind to SRSF1 and SRSF6 (Figure 2B). Agarose electrophoresis and Sanger sequencing of RT-PCR products confirmed that the c.1620C>T produced a band consistent with a transcript that only contains vector exons SD and SA, and c.3888C>T produced the expected full-length transcript (656 nt) with exons 18 and 19 and a 453-nt transcript with exon 18 skipping (25%) (Figures 2C,D). In four repeated experiments, using grayscale value analysis, the c.1620C>T resulted in exon 4 (164 bp) being skipped completely, and the c.3888C>T increased the proportion of abnormal splicing by 25% compared with the wild type (Figure 2C).

### Evaluation of 46 Synonymous Variants Predicted to Create ESS Motif

**FIGURE 3:**
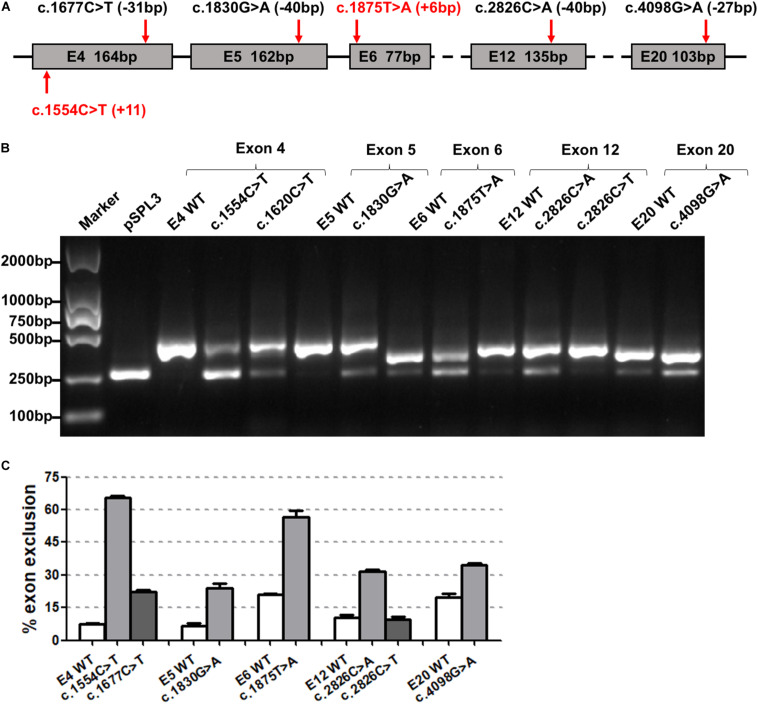
The effect of six synonymous variants predicted to create ESS motif on *ATP7B* pre-mRNA splicing. **(A)** The exonic localization of the six synonymous variants in the *ATP7B* gene. Variants marked in red are close to the junction of the intron and exon. **(B)** Agarose electrophoretic results of RT-PCR products of the six variants (c.1554C>T, c.1677C>T, c.1830G>A, c.1875T>A, c.2826C>A, and c.4098G>A) and corresponding wild types. **(C)** The grayscale value analysis shows that these six variations produced more abnormal splicing compared with the wild type: c.1554C>T increased the proportion of abnormal splicing by 58.15%, c.1677C>T increased by 16.79%, c.1830G>A increased by 17.40%, c.1875T>A increased by 35.77%, c.2826C>A increased by 21.44%, and c.4098G>A increased by 14.79%. All data are represented as mean ± SEM (*n* = 4). Quantification of the aberrant splicing percentage in the graph was calculated by densitometric analyses of the gel-fractionated RT-PCR products as the percentage of exclusion (%) = [MUT band/(MUT band + WT band)] × 100. WT, wild type; MUT, mutant.

The 46 candidate variants in the second group were verified by *in vitro* minigene assays. The results indicated that six variants (c.1554C>T, c.1677C>T, c.1830G>A, c.1875T>A, c.2826C>A, and c.4098G>A) affected the pre-mRNA splicing of *ATP7B* (Figure 3). The specific locations of the six variants in the *ATP7B* gene are shown in Figure 3A, in which the c.1554C>T and c.1875T>A variants are located within 15 bp downstream of the exon and intron boundary. The electrophoretic results showed that these variants produced a mutant band with a transcript that only contains vector exons SD and SA (Figure 3B), which were all confirmed by Sanger sequencing of the RT-PCR products (Supplementary Figure 2). The grayscale value analysis showed that these variations produced more abnormal splicing compared with the wild type; c.1554C>T increased the proportion of abnormal splicing by 58.15%, c.1677C>T increased by 16.79%, c.1830G>A increased by 17.40%, c.1875T>A increased by 35.77%, c.2826C>A increased by 21.44%, and c.4098G>A increased by 14.79% (Figure 3C).

The two variants (c.1554C>T and c.1677C>T) were both located in exon 4; the c.1554C>T nearby the boundary of the exon (+11 bp) and intron had a greater percentage of exon skipping. c.2826C>A and c.2826C>T were in the same position (c.2826), but abnormal splicing was observed only when cytosine (C) was mutated into adenine (A).

### Evaluation of 21 Synonymous Variants Predicted to Affect Donor or Acceptor Sites

**FIGURE 4:**
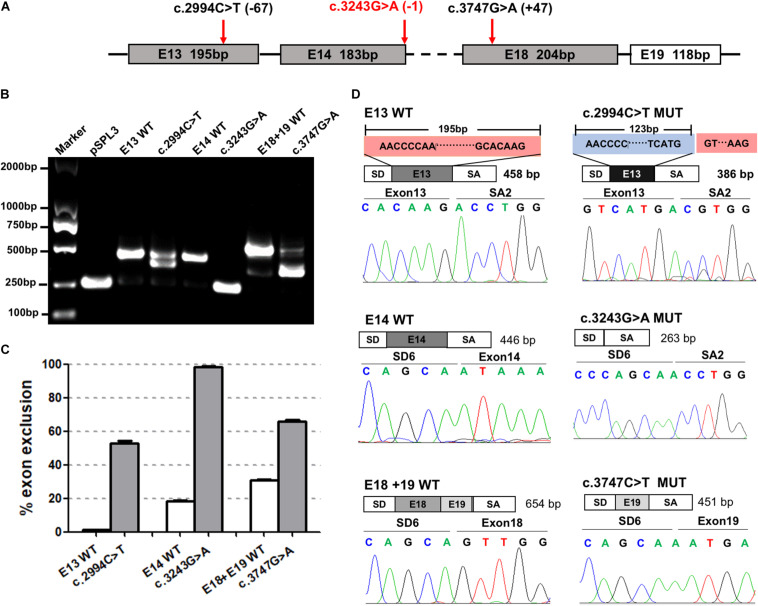
The effect of variants c.2994C>T, c.3243G>A, and c.3747G>A predicted to affect authentic donor or acceptor sites on *ATP7B* pre-mRNA splicing. **(A)** The exonic position of c.2994C>T, c.3243G>A, and c.3747G>A in the *ATP7B* gene. Variants marked in red are close to the junction of the intron and exon. **(B)** Agarose electrophoretic results of the RT-PCR products of c.2994C>T, c.3243G>A, c.3747G>A, and corresponding wild types. The RT-PCR band of the c.2994C>T mutant was ∼100 bp lower than the corresponding E13-WT band. The RT-PCR band of the c.3243G>A mutant was the same as the pSPL3 vector which only contains vector exons SD and SA. The RT-PCR band of the c.3747G>A WT produced the expected full-length transcript (656 bp) with exons 18 and 19, while the mutant showed one more minor band with a ∼450-bp length. **(C)** Grayscale value analysis shows that the c.2994C>T mutant produced an abnormal transcript with a percentage of 57.79% to total transcripts. The c.3243G>A mutant produced a complete exon 14 skipping (100%). The c.3747G>A mutant increased the exon 18 skipping by 34.93% compared with the wild type. All data are represented as mean ± SEM (*n* = 4). Quantification of the aberrant splicing percentage in the graph was calculated by densitometric analyses of the gel-fractionated RT-PCR products as the percentage of exclusion (%) = [MUT band/(MUT band + WT band)] × 100. **(D)** Sequencing chromatograms of the RT-PCR products amplified from the wild type and mutants. The sequencing of the c.2994C>T mutant band showed exon 13 was excluded (72 bp) compared with the 195-bp wildtype exon 13. The sequencing of the c.3243G>A and c.3747G>A mutants confirmed the skipping of exon 14 and exon 18, respectively. WT, wild type; MUT, mutant.

The minigene assays showed that three variants—c.2994C>T, c.3243G>A, and c.3747G>A—in the third group affected pre-mRNA splicing *in vitro* (Figure 4). The specific locations of the three variants in the *ATP7B* gene are detailed in Figure 4A. The c.2994C>T in exon 13 was predicted to activate an exonic cryptic donor site. The minigene assay showed that the c.2994C>T mutant produced an abnormal transcript which excluded 72 bp of exon 13 at the 3′ end, with a percentage of 57.79% to total transcripts (Figures 4B–D). c.3243G>A was located in a distinctive position, the last base of exon 14, and it was predicted probably to change canonical donor site (Figure 4A). The minigene verification showed that the variant led to the complete skipping of exon 14 (Figures 4B–D). c.3747G>A was selected because it predictably generated a new acceptor site, which unexpectedly showed exon 18 skipping in the minigene assay (Figures 4B–D). These results indicated that the c.3747G>A variant might disrupt the pre-mRNA splicing by altering some exonic splicing regulatory elements rather than creating a new acceptor site.

## Discussion

Next-generation sequencing has become the most effective tool for the diagnosis of WD, and more and more variants with uncertain clinical significance have been detected. Evaluating the pathogenicity of these uncertain variants is a great challenge to genetic diagnosis. Particularly, it is more difficult to assess synonymous mutations that do not change amino acids (Tiulpakov et al., 2020). Our previous study showed that the synonymous mutation c. 4014T>A in WD patient partially caused exon 19 exclusion in *ATP7B* pre-mRNA splicing (Wang et al., 2018). Most synonymous mutations are non-pathogenic mutations, but synonymous changes that affect splicing could lead to severe clinical manifestations (Byrne et al., 2009; Diao et al., 2014). In this study, the effect of synonymous variants in the *ATP7B* gene on pre-mRNA splicing was comprehensively analyzed in large quantities for the first time. A total of 253 rare synonymous variants were predicted by bioinformatics, and 85 variants were screened for minigene assays. Finally, our results showed that 11 variants had a different degree of impact on the pre-mRNA maturation of *ATP7B* (Table 1). Especially the three variants [c.1620C>T (Dong et al., 2016), c.1875T>A (Qian et al., 2019), and c.3243G>A (Qian et al., 2019)] have been reported in WD patients, which had not been functionally investigated. Our experimental results show that these three variants hinder the normal pre-mRNA splicing, in which c.1620C>T and c.3243G>A mutations completely caused exon skipping, and c.1875T>A increased abnormal splicing by 35%. All the three variants yielded a deleterious prediction score on disrupting authentic splice sites by two algorithms (HSF and NN Splicing).

Alterations in pre-mRNA splicing levels play a significant role in the development of human genetic diseases, including monogenic diseases as well as complex diseases. Studies have shown that mutations that bring about splicing dysfunction may account for 50% of all known mutations (Baralle and Buratti, 2017). The splicing process is strictly regulated and controlled by a series of proteins and SREs. The identification of splice sites depends on the conservative sequences around splice sites in introns and exons. Specific genetic variations can alter sequences such as splice sites, branching sites, or SREs, generating abnormal splicing (Wheway et al., 2019). The occurrence of these mutations may result in the abnormal use of 3′ or 5′ splice sites and may also lead to the abnormal inclusion of intron or exon skipping, resulting in the translation of proteins with abnormal amounts of amino acids (Anna and Monika, 2018).

ATP7B contains domains common to all P-type ATPases, including the six copper binding domains, the transmembrane domain with eight membrane spanning helices, the actuator domain, the phosphorylation domain, and the nucleotide-binding domain (Lutsenko et al., 2007; Telianidis et al., 2013). Prediction based on these functional domains indicated that complete skipping of exons 4, 6, and 20 will lead to early termination of translation and destructive changes in the ATP7B protein. The skip of exons 5, 12, and 14 would lead to the loss of the copper binding domain, the transmembrane region 5, and the phosphorylation domain, respectively. The truncation of exon 13 caused by c.2994C>T would lead to the deletion of amino acids at positions 1,997 to 1,020, which is close to the sixth transmembrane helix (including the copper binding sites). Previously, Wan et al. (2010) showed that the biological activity of the ATP7B mutant lacking exon 12 was 20% lower than that of wild-type ATP7B in *in vitro* studies, which might be responsible for a relatively milder disease condition and later onset age in WD patients. Further functional studies are needed to elucidate the deleteriousness of the absence of these functional domains caused by splicing variants.

The accuracy of bioinformatics analysis is limited. The use of a variety of bioinformatics methods for assessment combined with *in vitro* experimental studies can effectively increase the accuracy. RT-PCR with the RNA of carriers can directly reflect the effects of variants on splicing, but it is difficult to obtain samples from target tissues regarding the genes with low expression in peripheral blood and some exons are also too long to be amplified by RT-PCR (Bryksin and Matsumura, 2010). A large number of studies have shown that a minigene (pSPL3) can simulate exon splicing *in vitro* and can be used to analyze the splicing effect (Byrne et al., 2009; Acedo et al., 2015; Dal Mas et al., 2015; Takeuchi et al., 2015; Wang et al., 2018). In our previous study, we demonstrated that the ATP7B RT-PCR results from the peripheral blood of affected individuals were consistent with those of minigene assays *in vitro*, suggesting that a minigene assay can be used to verify the splicing of the *ATP7B* gene (Wang et al., 2018). We resoundingly constructed 14 wild-type Minigene_ATP7B_ex in this study. The corresponding wild-type plasmids of the first and the last exon (exon 1 and exon 21) were not constructed. Moreover, exon 2 and exons 8 and 9 could not be simulated successfully in the pSPL3 system, which may be due to the existence of key splicing regulatory elements in the deep intron region (Garanto et al., 2019).

Several limitations of this study should be mentioned. Firstly, splicing is a very complex process, which can vary between different tissues and different cell types. Thus, human samples from the tissues of interest are always the best way to evaluate the splicing effect of variants. However, the *ATP7B* gene is mainly expressed in hepatocytes, and it is difficult to obtain liver tissue *in vivo*. What is more, the frameshift mutations could trigger nonsense-mediate decay (NMD)-driven degradation of the mutant mRNA (Zarraga et al., 2011; Gong and Zhou, 2018). NMD assay was not conducted in our study. Further studies are required to determine whether the abnormal mRNA produced by these synonymous variants in *ATP7B* is subject to NMD.

## Conclusion

In conclusion, we prioritized 253 naturally occurring rare *ATP7B* synonymous variants by computational tools and subsequent functional assessment using a minigene splicing assay, which provides evidences that 11 variants alter pre-mRNA splicing *in vitro*. However, the specific mechanism underlying these splicing disruptions needs to be further studied. We expect that the application of these methodologies could provide reliable experimental basis for genetic diagnosis and genetic counseling of WD patients with these synonymous variations.

## Data Availability Statement

All data generated or analyzed during this study are included in this published article.

## Author Contributions

XZ and WZ performed bioinformatics predictions and minigene assays and were major contributors in writing the manuscript. CW and LW screened the candidate variations. ZJ revised the manuscript. YJ, ZL, and BZ designed the study, guided the writing of the manuscript, and critically reviewed the manuscript. All the authors read and approved the final manuscript.

## Conflict of Interest

The authors declare that the research was conducted in the absence of any commercial or financial relationships that could be construed as a potential conflict of interest.

## Footnotes

1 http://www.hgmd.cf.ac.uk/ac/index.php

2 http://exac.broadinstitute.org

3 https://gnomad.broadinstitute.org/

4 http://www.umd.be/HSF/

5 http://rulai.cshl.edu/cgi-bin/tools/ESE3/esefinder.cgi

## Supplementary Material

Supplementary Figure 1 A schematic representation of the pSPL3 vector.

Supplementary Figure 2 Sanger sequencing results of the RT-PCR products of the 14 successfully constructed wild-type Minigene_ATP7B_ex plasmids.

Supplementary Table 1 Basic information of 253 synonymous variants in the ATP7B gene.

Supplementary Table 2 Primers for amplification and cloning of fragments containing individual exons.

Supplementary Table 3 Primers for site-directed mutations.
