# Supplementary material for: A Comprehensive Analysis and Splicing Characterization of Naturally Occurring Synonymous Variants in the ATP7B Gene
Source: Front Genet. 2021 Feb 25;11:592611. doi: 10.3389/fgene.2020.592611 (PMC7947925; doi:10.3389/fgene.2020.592611)
Supplement: Supplementary Figure 1 — A schematic representation of the pSPL3 vector. [file Data_Sheet_1.PDF]

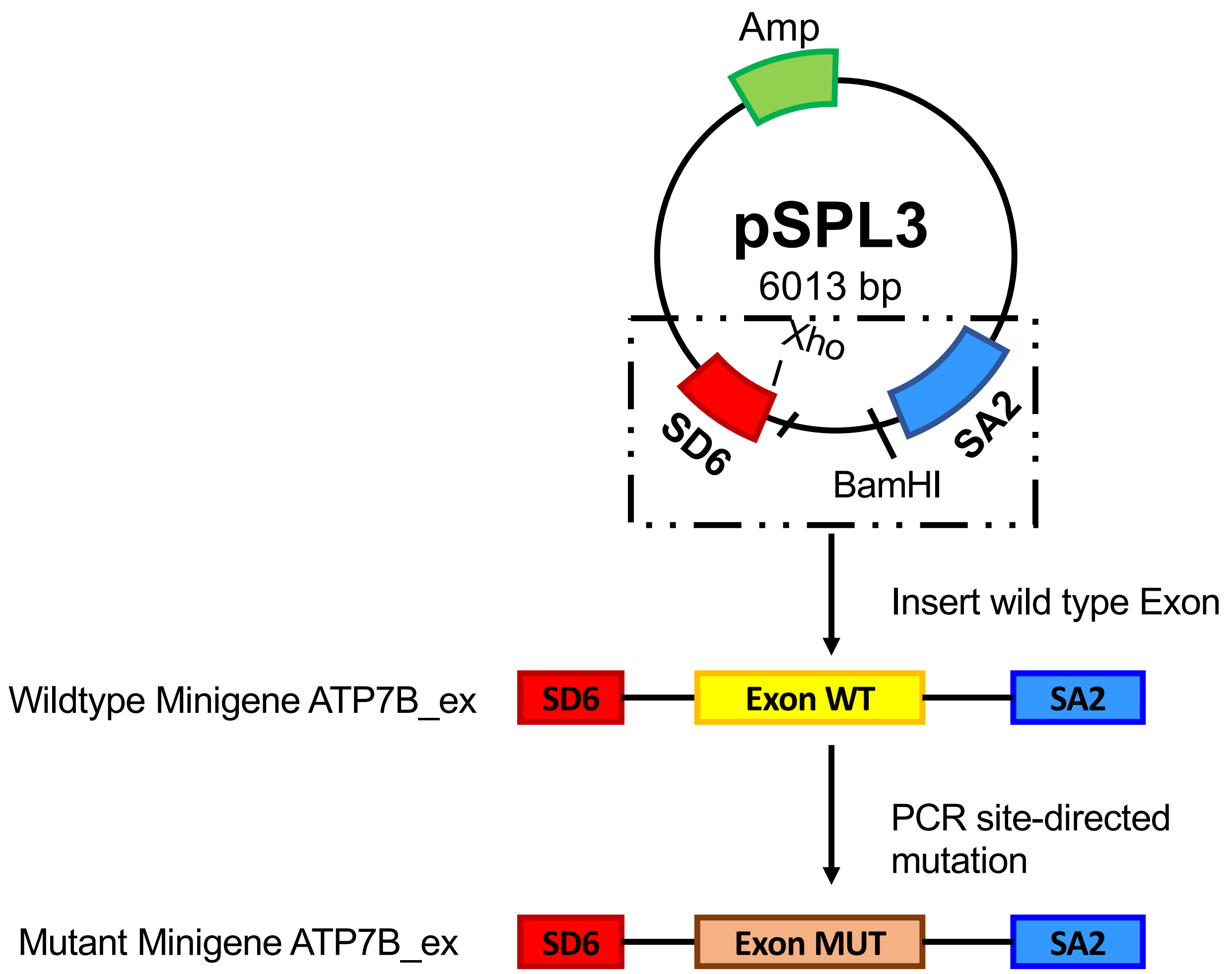

Figure S1 A schematic representation of pSPL3 vector. The minigene splicing assay based on the pSPL3 exon trapping vector. A, The pSPL3 vector contains 2 exons SD and SA, and a functional intron, all of the indicated fragments were separately cloned into the XhoI and BamHI cloning sites of the pSPL3 vector. SD6 and SA2 primers were designed for RT-PCR amplification of cDNA sequences generated by transfected HEK293T cells. 85 different mutant Minigene ATP7B_ex capture plasmids were constructed by PCR-mediated in vitro site-directed mutagenesis.

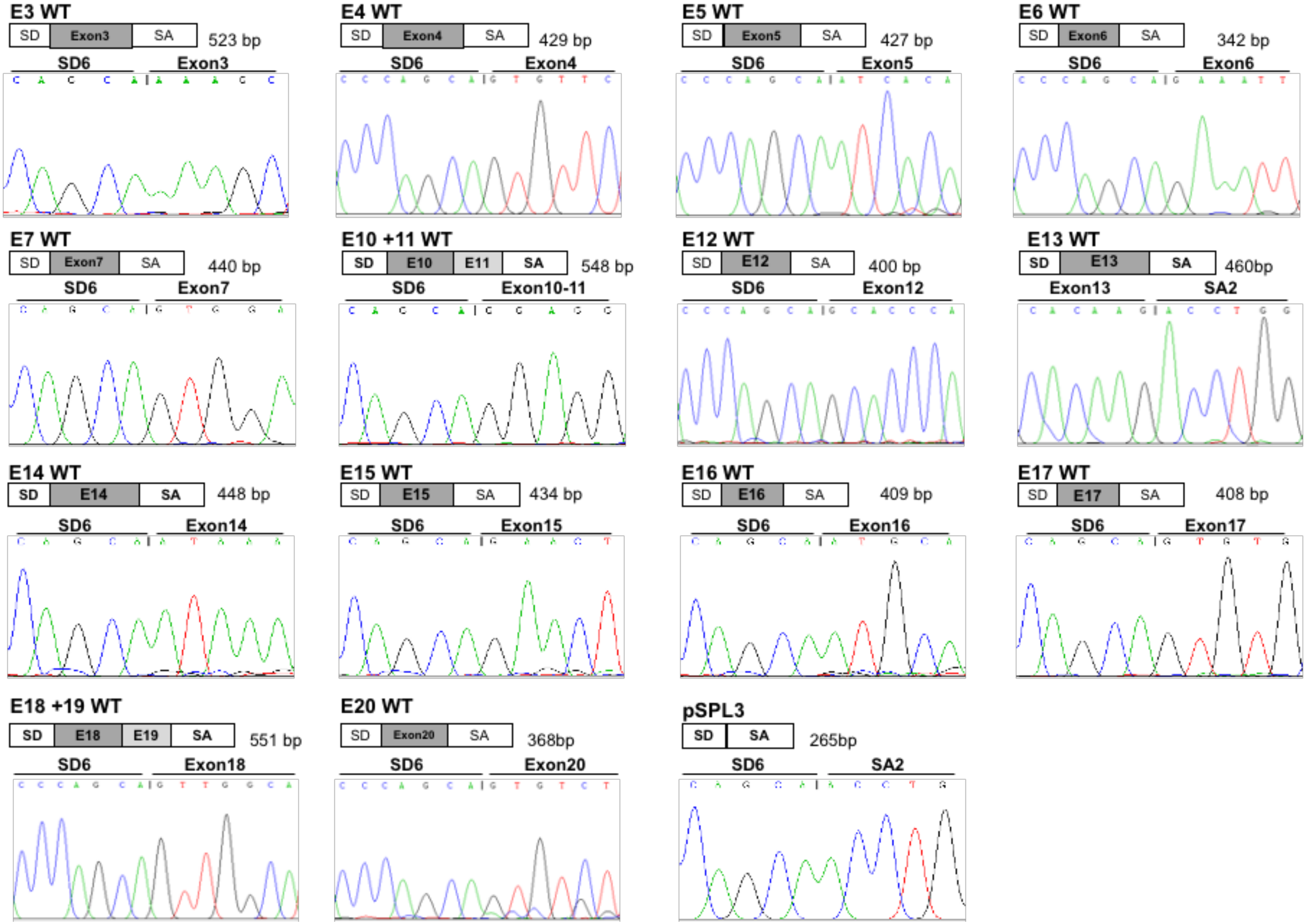

Figure S2 Sanger sequencing results of RT-PCR products of the 14 successfully constructed wildtype Minigene_ATP7B_ex plasmids.
