# Supplementary material for: A Comprehensive Analysis and Splicing Characterization of Naturally Occurring Synonymous Variants in the ATP7B Gene
Source: Front Genet. 2021 Feb 25;11:592611. doi: 10.3389/fgene.2020.592611 (PMC7947925; doi:10.3389/fgene.2020.592611)
Supplement: Supplementary Table 3 — Primers for site-directed mutations. [file Table_3.DOC]

**Table S3. Primers for site-directed mutations**

| **Number** | **DNA variants** |  | **Sequence 5’→3’** |
| --- | --- | --- | --- |
| 1 | c.1362A>G | F | ACACCTACgTCTGTGCAGGAAGTGGCTCCCCA |
|  |  | R | TGCACAGAcGTAGGTGTACCATCTGTAGTTTGCACC |
| 2 | c.1416G>A | F | CATGCCCCaGACATCTTGGCAAAGTCCCCACA |
|  |  | R | AAGATGTCtGGGGCATGGTTTGCAGGGAGCCT |
| 3 | c.1449A>G | F | AATCAACCAGgGCAGTGGCACCGCAGAAGTGC |
|  |  | R | CACTGCcCTGGTTGATTGTGGGGACTTTGCCA |
| 4 | c.1458A>T | F | tCCGCAGAAGTGCTTCTTACAGATCAAAGGCA |
|  |  | R | AGAAGCACTTCTGCGGaGCCACTGCTCTGGTTGATTGTG |
| 5 | c.1524G>A | F | CATAGAAAGaAATCTGCAGAAAGAAGCTGGTAAGA |
|  |  | R | GCAGATTtCTTTCTATGTTAGACACACAGGATGCA |
| 6 | c.1554C>T | F | GTTCTCTCtGTGTTGGTTGCCTTGATGGCAGG |
|  |  | R | ACCAACACaGAGAGAACACCTGGAACCATCAG |
| 7 | c.1605G>A | F | GACCCAGAaGTCATCCAGCCCCTCGAGATAGC |
|  |  | R | TGGATGACtTCTGGGTCATACTTGATCTCTGCC |
| 8 | c.1620C>T | F | AGCCCCTtGAGATAGCTCAGTTCATCCAGGACC |
|  |  | R | AGCTATCTCaAGGGGCTGGATGACCTCTGGGT |
| 9 | c.1662A>G | F | TTTGAGGCAGCgGTCATGGAGGACTACGCAGGC |
|  |  | R | ATGACcGCTGCCTCAAAACCCAGGTCCTGGAT |
| 10 | c.1677C>T | F | ATGGAGGACTAtGCAGGCTCCGATGGCAACAT |
|  |  | R | CCTGCaTAGTCCTCCATGACTGCTGCCTCAAA |
| 11 | c.1716G>T | F | ATCACAGGtATGACCTGCGCGTCCTGTGTCCA |
|  |  | R | CAGGTCATaCCTGTGATCTGCAACACAGGATG |
| 12 | c.1725C>T | F | TCACAGGGATGACCTGtGCGTCCTGTGTCCACAACATAG |
|  |  | R | aCAGGTCATCCCTGTGATCTGCAACACAGGAT |
| 13 | c.1761G>A | F | CCAAACTCACaAGGACAAATGGCATCACTTATGC |
|  |  | R | TGTCCTtGTGAGTTTGGACTCTATGTTGTGGA |
| 14 | c.1788C>T | F | TATGCCTCtGTTGCCCTTGCCACCAGCAAAGC |
|  |  | R | AGGGCAACaGAGGCATAAGTGATGCCATTTGT |
| 15 | c.1803C>T | F | CTTGCCACtAGCAAAGCCCTTGTTAAGTTTGA |
|  |  | R | GCTTTGCTaGTGGCAAGGGCAACGGAGGCATA |
| 16 | c.1815T>G | F | CAAAGCCCTgGTTAAGTTTGACCCGGAAATTATCG |
|  |  | R | ACTTAACcAGGGCTTTGCTGGTGGCAAGGGCA |
| 17 | c.1830G>A | F | GACCCaGAAATTATCGGTCCACGGGATATTAT |
|  |  | R | CCGATAATTTCtGGGTCAAACTTAACAAGGGCTTT |
| 18 | c.1839C>T | F | AATTATtGGTCCACGGGATATTATCAAAATTA |
|  |  | R | CCCGTGGACCaATAATTTCCGGGTCAAACTTAACAA |
| 19 | c.1875T>A | F | TaGGCTTTCATGCTTCCCTGGCCCAGAGAAAC |
|  |  | R | GGAAGCATGAAAGCCtATTTCCTTGTCATTAAAAAGAGAGGG |
| 20 | c.1965G>A | F | TCTTTCCTaTGCAGCCTGGTGTTTGGCATCCC |
|  |  | R | AGGCTGCAtAGGAAAGACTTCTTCCACCTGGA |
| 21 | c.1998C>T | F | CCCTGTCATGGCtTTAATGATCTATATGCTGATACCCAGC |
|  |  | R | TTAAaGCCATGACAGGGATGCCAAACACCAGG |
| 22 | c.2082T>C | F | GGACTGTCCATcCTAAATCTCATCTTCTTTATCTTGTGTACC |
|  |  | R | TTTAGgATGGACAGTCCTGGAATGATGTTGTG |
| 23 | c.2463C>T | F | CAAGTCCCtATGGAGCTGGTGCAGCGGGGCGA |
|  |  | R | AGCTCCATaGGGACTTGCTCCTCCCTGCAACA |
| 24 | c.2484C>T | F | CGGGGtGATATCGTCAAGGTGGTCCCTGGGGG |
|  |  | R | TTGACGATATCaCCCCGCTGCACCAGCTCCAT |
| 25 | c.2511A>G | F | GGgAAGTTTCCAGTGGATGGGAAAGTCCTGGA |
|  |  | R | TCCACTGGAAACTTcCCCCCAGGGACCACCTTG |
| 26 | c.2544C>T | F | CCTGGAAGGtAATACCATGGCTGATGAGTCCC |
|  |  | R | TGGTATTaCCTTCCAGGACTTTCCCATCCACT |
| 27 | c.2667C>T | F | CTACCCAtGTGGGCAATGACACCACTTTGGCT |
|  |  | R | ATTGCCCACaTGGGTAGCTTTAATGAGCACAGAG |
| 28 | c.2679C>T | F | GGGCAATGAtACCACTTTGGCTCAGATTGTGAA |
|  |  | R | AAGTGGTaTCATTGCCCACGTGGGTAGCTTTA |
| 29 | c.2712A>G | F | TGAAACTGGTGGAgGAGGCTCAGATGTCAAAGGTAATG |
|  |  | R | CTCcTCCACCAGTTTCACAATCTGAGCCAAAG |
| 30 | c.2790C>A | F | TaATGTCAACTTTGACGTTGGTGGTATGGATT |
|  |  | R | CGTCAAAGTTGACATtATGATGATAAATGGGACAAAATATCC |
| 31 | c.2811G>A | F | GACGTTGGTaGTATGGATTGTAATCGGTTTTATCGA |
|  |  | R | TCCATACtACCAACGTCAAAGTTGACATGATG |
| 32 | c.2826C>A | F | GTATGGATTGTAATaGGTTTTATCGATTTTGGTGTTGTTC |
|  |  | R | CCtATTACAATCCATACCACCAACGTCAAAGT |
| 33 | c.2826C>T | F | GTATGGATTGTAATtGGTTTTATCGATTTTGGTGTTGTTC |
|  |  | R | CCaATTACAATCCATACCACCAACGTCAAAGT |
| 34 | c.2892A>C | F | CAGACcGAGGTGATCATCCGGTTTGCTTTCCA |
|  |  | R | ATGATCACCTCgGTCTGGGAGATGTGCTTGTTGG |
| 35 | c.2931G>T | F | TCCATCACtGTGCTGTGCATTGCCTGCCCCTG |
|  |  | R | CACAGCACaGTGATGGACGTCTGGAAAGCAAA |
| 36 | c.2931G>A | F | TCCATCACtGTGCTGTGCATTGCCTGCCCCTG |
|  |  | R | CACAGCACtGTGATGGACGTCTGGAAAGCAAA |
| 37 | c.2955C>T | F | ATTGCCTGCCCCTGtTCCCTGGGGCTGGCCACG |
|  |  | R | GAaCAGGGGCAGGCAATGCACAGCACCGTGAT |
| 38 | c.2964G>T | F | TCCCTGGGtCTGGCCACGCCCACGGCTGTCAT |
|  |  | R | GTGGCCAGaCCCAGGGAGCAGGGGCAGGCAAT |
| 39 | c.2979G>C | F | ACGCCCACcGCTGTCATGGTGGGCACCGGGGT |
|  |  | R | ATGACAGCgGTGGGCGTGGCCAGCCCCAGGGA |
| 40 | c.2985C>T | F | ACGGCTGTtATGGTGGGCACCGGGGTGGCCGC |
|  |  | R | CCCACCATaACAGCCGTGGGCGTGGCCAGCCC |
| 41 | c.2994C>T | F | TCATGGTGGGtACCGGGGTGGCCGCGCAGAA |
|  |  | R | GCGGTaCCCACCATGACAGCCGTGGGCGTGG |
| 42 | c.2997C>T | F | TGTCATGGTGGGCACtGGGGTGGCCGCGCAGAAC |
|  |  | R | CaGTGCCCACCATGACAGCCGTGGGCGTGGCC |
| 43 | c.2997C>G | F | TGTCATGGTGGGCACgGGGGTGGCCGCGCAGAAC |
|  |  | R | CcGTGCCCACCATGACAGCCGTGGGCGTGGCC |
| 44 | c.3006C>T | F | GCtGCGCAGAACGGCATCCTCATCAAGGGAGG |
|  |  | R | ATGCCGTTCTGCGCaGCCACCCCGGTGCCCACC |
| 45 | c.3033A>T | F | TCATCAAGGGtGGCAAGCCCCTGGAGATGGCG |
|  |  | R | CTTGCCaCCCTTGATGAGGATGCCGTTCTGCG |
| 46 | c.3043C>T | F | tTGGAGATGGCGCACAAGGTCAGCCTGTAGCA |
|  |  | R | TTGTGCGCCATCTCCAaGGGCTTGCCTCCCTTGATG |
| 47 | c.3090C>T | F | AGACTGGtACCATTACCCATGGCGTCCCCAGG |
|  |  | R | GGTAATGGTaCCAGTCTTGTCAAACATCACAGTCT |
| 48 | c.3105C>T | F | ATTACCCATGGtGTCCCCAGGGTCATGCGGGT |
|  |  | R | GGGACaCCATGGGTAATGGTGCCAGTCTTGTC |
| 49 | c.3114G>A | F | AGaGTCATGCGGGTGCTCCTGCTGGGGGATGT |
|  |  | R | AGCACCCGCATGACtCTGGGGACGCCATGGGTA |
| 50 | c.3129C>G | F | ATGCGGGTGCTgCTGCTGGGGGATGTGGCCAC |
|  |  | R | AGCAGcAGCACCCGCATGACCCTGGGGACGCC |
| 51 | c.3129C>T | F | ATGCGGGTGCTtCTGCTGGGGGATGTGGCCAC |
|  |  | R | AGCAGaAGCACCCGCATGACCCTGGGGACGCC |
| 52 | c.3150A>G | F | ACgCTGCCCCTCAGGAAGGTTCTGGCTGTGGT |
|  |  | R | TTCCTGAGGGGCAGcGTGGCCACATCCCCCAGC |
| 53 | c.3160A>C | F | AGTGCCCCTCcGGAAGGTTCTGGCTGTGGTGGGGACT |
|  |  | R | AGAACCTTCCgGAGGGGCAGTGTGGCCACATCCCCATC |
| 54 | c.3169C>T | F | GGAAGGTTtTGGCTGTGGTGGGGACTGCGGAG |
|  |  | R | CACAGCCAaAACCTTCCTGAGGGGCAGTGTGG |
| 55 | c.3189G>A | F | CaGAGGCCAGCAGTGAACACCCCTTGGGCGTG |
|  |  | R | TTCACTGCTGGCCTCtGCAGTCCCCACCACAGCC |
| 56 | c.3207C>T | F | AGTGAACAtCCCTTGGGCGTGGCAGTCACCAA |
|  |  | R | CCCAAGGGaTGTTCACTGCTGGCCTCCGCAGT |
| 57 | c.3243G>A | F | AGAaGTACGTGGACTTGGGCGTGGCCCTGCCC |
|  |  | R | CAAGTCCACGTACtTCTTTACAGTATTTGGTGACTGCCA |
| 58 | c.3261C>G | F | ACAGAGACgTTGGGATACTGCACGGACTTCCA |
|  |  | R | TATCCCAAcGTCTCTGTTCCAAGTTCCTGGGA |
| 59 | c.3276G>A | F | ACTTCCAGaCAGTGCCAGGCTGTGGAATTGGG |
|  |  | R | TGGCACTGtCTGGAAGTCCGTGCAGTATCCCA |
| 60 | c.3336C>T | F | ACGTGGAAGGCATtCTGGCCCACAGTGAGCGC |
|  |  | R | CAGaATGCCTTCCACGTTGCTGACTTTGCACC |
| 61 | c.3366A>C | F | TTTGAGTGCcCCGGCCAGTCACCTGAATGAGG |
|  |  | R | TGGCCGGgGCACTCAAAGGGCGCTCACTGTGG |
| 62 | c.3366A>G | F | TTTGAGTGCgCCGGCCAGTCACCTGAATGAGG |
|  |  | R | TGGCCGGcGCACTCAAAGGGCGCTCACTGTGG |
| 63 | c.3369G>A | F | TTTGAGTGCACCaGCCAGTCACCTGAATGAGGC |
|  |  | R | TGGCtGGTGCACTCAAAGGGCGCTCACTGTGG |
| 64 | c.3402C>T | F | AGCCTTCCtGCAGAAAAAGGTATTGCTGGCTT |
|  |  | R | TTTTCTGCaGGAAGGCTGCCAGCCTCATTCAG |
| 65 | c.3462G>A | F | AACCGTGAGTGGCTaAGGCGCAACGGTTTAACCA |
|  |  | R | CTtAGCCACTCACGGTTTCCAATCAGCACAGA |
| 66 | c.3471C>T | F | AGGCGCAAtGGTTTAACCATTTCTAGCGATGTCA |
|  |  | R | GTTAAACCaTTGCGCCTCAGCCACTCACGGTT |
| 67 | c.3489C>T | F | GTCAGTGAtGCTATGACAGACCACGAGATGAAA |
|  |  | R | GTCATAGCaTCACTGACATCGCTAGAAATGGTT |
| 68 | c.3501C>T | F | GTCAGTGAtGCTATGACAGACCACGAGATGAAA |
|  |  | R | GTCATAGCaTCACTGACATCGCTAGAAATGGTT |
| 69 | c.3516C>T | F | GACAGACCAtGAGATGAAAGGACAGACAGCCAT |
|  |  | R | TCATCTCaTGGTCTGTCATAGCGTCACTGACA |
| 70 | c.3588C>T | F | ATCGCAGAtGCTGTCAAGCAGGAGGCTGCCCT |
|  |  | R | TTGACAGCaTCTGCGATTGCGATCATCCCACA |
| 71 | c.3624G>A | F | TGTGCACACaCTGCAGAGCATGGGTGTGGACG |
|  |  | R | TCTGCAGtGTGTGCACAGCCAGGGCAGCCTCC |
| 72 | c.3645C>T | F | GGTGTGGAtGTGGTTCTGATCACGGGGGACAA |
|  |  | R | AGAACCACaTCCACACCCATGCTCTGCAGCGT |
| 73 | c.3732G>T | F | GTGCTtCCTTCGCACAAGGTGGCCAAGGTCCA |
|  |  | R | TTGTGCGAAGGaAGCACCTCTGCAAAGACTTTGTT |
| 74 | c.3747G>A | F | CACAAGGTaGCCAAGGTCCAGGAGCTCCAGAA |
|  |  | R | ACCTTGGCtACCTTGTGCGAAGGCAGCACCTC |
| 75 | c.3783A>G | F | AAAGGGAAGAAgGTCGCCATGGTGGGGGATGG |
|  |  | R | GCGACcTTCTTCCCTTTATTCTGGAGCTCCTG |
| 76 | c.3786C>T | F | GAAGAAAGTtGCCATGGTGGGGGATGGGGTCA |
|  |  | R | CCATGGCaACTTTCTTCCCTTTATTCTGGAGCT |
| 77 | c.3864G>A | F | ACaGATGTGGCCATCGAGGCAGCCGACGTCGT |
|  |  | R | TCGATGGCCACATCtGTGCCGGTGCCAATGGCC |
| 78 | c.3888C>T | F | AGCCGAtGTCGTCCTTATCAGAGTGAGCGTGG |
|  |  | R | TAAGGACGACaTCGGCTGCCTCGATGGCCACA |
| 79 | c.3894C>T | F | GACGTCGTtCTTATCAGAGTGAGCGTGGCTGC |
|  |  | R | CTGATAAGaACGACGTCGGCTGCCTCGATGGC |
| 80 | c.4014T>C | F | TTGGGATACCCATcGCAGCAGGTAGGCAGCTCTTA |
|  |  | R | TGCgATGGGTATCCCAACCAGGTTATAAATCA |
| 81 | c.4014T>A | F | ATACCCATaGCAGCAGGTAGGCAGCTCTTACC |
|  |  | R | CCTGCTGCtATGGGTATCCCAACCAGGTTATAA |
| 82 | c.4038C>T | F | ATGCCCATtGGCATTGTGCTGCAGCCCTGGAT |
|  |  | R | ACAATGCCaATGGGCATGAAGACACCTGGGGA |
| 83 | c.4092G>T | F | TCCTCTGTtTCTGTGGTGCTCTCATCCCTGCA |
|  |  | R | ACCACAGAaACAGAGGAGGCTGCCATGGCCGC |
| 84 | c.4098G>A | F | GTGTCTGTaGTGCTCTCATCCCTGCAGCTCAA |
|  |  | R | GAGAGCACtACAGACACAGAGGAGGCTGCCAT |
| 85 | c.4110C>T | F | CTCTCATCtCTGCAGCTCAAGTGGTGAGTCCC |
|  |  | R | AGCTGCAGaGATGAGAGCACCACAGACACAGAG |

*Minuscule indicate the site of the mutation.
